# Influence of Screw Length and Bone Quality on Pedicle Screw Anchorage Under Cyclic Fatigue Loading

**DOI:** 10.1177/21925682261442309

**Published:** 2026-04-13

**Authors:** Friederike Eva Roch, Betül Tekin, Katharina Jäckle, Marc-Pascal Meier, Friederike Klockner, Hassan Awan Malik, Wolfgang Lehmann, Lukas Weiser, Paul Jonathan Roch

**Affiliations:** 1 Department of Trauma Surgery, Orthopaedics and Plastic Surgery, University of Göttingen, Göttingen, Germany

**Keywords:** pedicle screw, hold, pedicle, vertebra, osteoporosis

## Abstract

**Study Design:**

Cadaveric biomechanical analysis.

**Objectives:**

The specific vertebral region that contributes most critically to pedicle screw fixation remains controversial. This study evaluates screw anchorage under cyclic fatigue loading to determine how bone quality affects the relative contributions of the pedicle and vertebral body to stability.

**Methods:**

Twelve human lumbar vertebrae (L1–L4) from donors aged 71.3 ± 14.2 years were selected, including six with normal bone density (>120 mg/cm^3^) and six osteoporotic (<80 mg/cm^3^). Each vertebra was bilaterally instrumented with the largest self-tapping polyaxial screws fitting the inner cortical diameter. One pedicle received a 35 mm screw, and the contralateral pedicle received the longest screw without cortical breach. Screw side and specimen order were randomized. Sinusoidal cranial-caudal loading at 0.5 Hz was applied, starting at 100 N and increasing by 1 *N* per cycle, until 5.4 mm screw head displacement (∼20°) was reached.

**Results:**

In healthy vertebrae, mean fatigue loads were similar for short (315.6 ± 148.7 *N*) and long screws (309.0 ± 138.3 *N*). In osteoporotic bone, long screws (230.9 ± 55.0 *N*) showed significantly greater fatigue resistance than short screws (175.1 ± 45.5 *N*; *P* = .045). Short screws in osteoporotic vertebrae also failed after fewer cycles than long screws in osteoporotic vertebrae (*P* = .047) and short screws in healthy bone vertebrae (*P* = .049).

**Conclusions:**

In healthy vertebrae, screw anchorage is mainly pedicular, and extending screw length into the vertebral body adds no benefit. In osteoporotic bone, overall fixation strength declines, but vertebral body contribution increases, so longer screws provide significantly greater stability, whereas shorter screws remain adequate in healthy bone.

## Introduction

The overall mechanical integrity of a posterior spinal fixation system is dictated by the anchorage capacity of its pedicle screws, which is determined by how effectively the screw threads interlock with the surrounding vertebral bone.^1^

Pedicle screw pull-out resistance is influenced by a variety of biomechanical and anatomical factors. These include the screw’s trajectory, insertion depth, and positioning relative to cortical bone (eg, bicortical purchase), as well as the use of techniques such as tapping, pilot hole preparation, and screw reinsertion.^2,3,4,5^ The structural design of the screw—specifically its outer diameter, thread pitch, depth, and shape—also plays a significant role.^1^

Although the pedicle screw is named after its point of entry, the vertebral region that provides the predominant contribution to screw holding strength remains a matter of debate. Misenhimer, Peek, Wiltse, Rothman and Widell^6^ demonstrated that screws whose diameters exceed the pedicle width induce plastic deformation of the pedicle before cortical breach or cut-out, suggesting that the pedicle cortex itself supplies limited support for oversized implants. Defino and Vendrame^7^ analyzing computed-tomography sections, likewise reported that cancellous rather than cortical bone is critical for screw retention within the pedicle.

In an specimen-based study, Cornaz, Farshad and Widmer^8^ inserted 10 mm pedicle-screw segments into sliced pedicles and vertebral bodies and subjected them to axial loading. Under small cyclic amplitudes, the pedicle and vertebral body contributed equally to fixation regardless of bone quality. As loading amplitudes rose, the pedicle’s contribution increased disproportionately, and this effect became even more pronounced in osteoporotic bone.

Hirano, Hasegawa, Takahashi, Uchiyama, Hara, Washio, Sugiura, Yokaichiya and Ikeda^9^ evaluated pedicle screw stability under caudocephalad and pull-out loading in vertebrae with and without the vertebral body. They found that approximately 80% of the caudocephalad stiffness and 60% of the pull-out strength of the pedicle screw depended on the pedicle rather than the vertebral body, with bone mineral density showing no significant influence. Although several metrics have been proposed to quantify screw stability, insertion torque and single-cycle pull-out tests do not replicate physiological loading conditions and correlate poorly with clinical screw loosening. In contrast, cyclic fatigue (“toggle”) testing is widely regarded as the preclinical gold standard for evaluating pedicle screw performance.

The present investigation therefore employs cyclic fatigue testing to compare short and long pedicle screws in osteoporotic and non-osteoporotic vertebrae. Its primary aims are (i) to identify the vertebral region that provides maximal anchorage for the pedicle screw and (ii) to determine whether this locus varies with bone quality.

## Material and Methods

### Specimen

Twelve human lumbar vertebral bodies (L1–L4) were harvested from donors aged 53-94 years (71.3 ± 14.2 years): six with normal bone density (>120 mg/cm^3^) and six osteoporotic (<80 mg/cm^3^). Specimens were sealed in plastic bags and stored below −20°C. CT scans (SOMATOM definition AS+, Siemens, Munich, Germany) were used to exclude fractures or pathologies. Volumetric bone mineral density (vBMD) was measured using CliniQCT® software (Mindways Software, Inc, USA), with asynchronous calibration against a bone phantom.

CliniQCT® determined vBMD through five steps: (1) selecting L1–L4 on mid-sagittal slices, (2) isolating vertebrae on mid-axial slices, (3) spatial alignment, (4) automated ROI selection, and (5) manual ROI adjustment 1 mm from the cortex. CT parameters: 120 kVp, 500 mm FOV, 1.000 uniformity correction, I30f/B30s filter, 1 mm slice thickness. vBMD was reported in mg/cm^3^.

**Figure 1.**
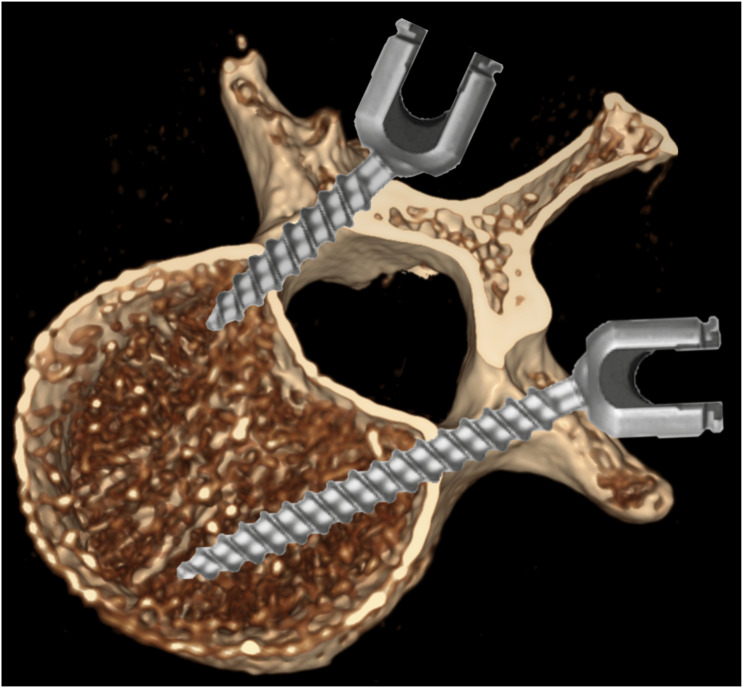
Schematic illustration of pedicle screw insertion. The screw diameter in each pedicle was maximized to the inner cortical diameter. On one side, the longest monocortical screw (ie, not breaching the anterior cortex) was inserted, whereas the contralateral side received a screw of fixed length (35 mm)

Specimens were thawed overnight and soft tissues removed. Vertebrae were kept moist with Ringer solution. Both pedicles were instrumented with the largest possible self-tapping polyaxial screws (Expedium, Synthes, USA), selected according to the smallest inner cortical diameter of the pedicle as determined by CT imaging. One pedicle received the longest screw possible without cortical breach, as determined by CT imaging; the other always received a 35 mm screw. Pedicle screw placement was performed under direct visual control, as the vertebrae were completely cleared of surrounding soft tissue. The screw trajectory was initially created using an awl and subsequently palpated to confirm an intact pedicle channel before instrumentation. Pedicle screws were then inserted along the prepared trajectory. All procedures were performed by senior spine surgeons. Additional fluoroscopic guidance was not used, as it was not considered to provide further benefit under these controlled experimental conditions with direct visual access. The screw side and the order of specimens were randomized (Table 1, Figure 1).

**Table 1.** Specimen Characteristics

	Donor	Specimen	Age (y)	Gender (F/M)	Level	BMD (mg/cm^3^)	Screws					
							Short screws			Long screws		
							*Side*	*Diameter*	*Length*	*Side*	*Diameter*	*Length*
Healthy	1	1	53	F	L2	120.4	L	5	35	R	5	45
	1	2	53	F	L3	125.8	R	6	35	L	6	45
	1	3	53	F	L4	124.1	R	7	35	L	7	45
	2	1	64	M	L1	134.7	R	8	35	L	8	50
	2	2	64	M	L3	137.7	L	10	35	R	10	55
	3	1	60	F	L2	147.6	L	5	35	R	5	50
Osteoporotic	1	1	82	F	L1	60.2	L	5	35	R	5	50
	1	2	82	F	L2	58.8	R	6	35	L	6	50
	1	3	82	F	L3	55.1	R	8	35	L	8	50
	2	1	84	M	L1	89.8	R	7	35	L	7	50
	2	2	84	M	L4	27.4	L	10	35	R	10	50
	3	1	94	M	L2	77.8	L	5	35	R	5	55

### Experimental Setup

**Figure 2.**
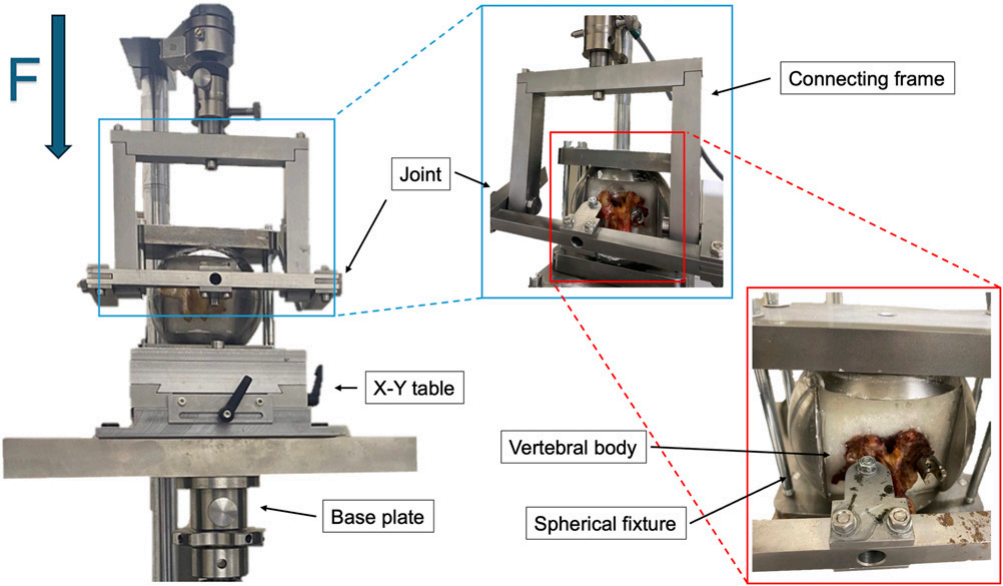
Experimental setup. Horizontal alignment of the screw was established by placing each specimen within a spherical potting fixture seated on an x–y table. The screw head was coupled to a hinge-equipped connecting frame whose pivot—coaxial with the head—restricted movement to one plane orthogonal to the screw’s shaft. After these adjustments, a vertically directed axial force was applied

A detailed description of the experimental design can be found in earlier work; it is briefly summarized here for the sake of completeness.^10,11^ Screws were inserted parallel to the endplate. Specimens were embedded anteriorly in cold polymer (Weitur-Press Standard, Weithas, Germany), leaving posterior arches and pedicles exposed. Fixtures were mounted on an x-y table of a Zwick materials tester (145.660 Z020/TND, Zwick, Ulm, Germany), positioning screws horizontally. The screw heads were connected to the actuator via a 2 cm rod (Expedium 5.5 mm, Synthes, USA) using set screws, with a rotational axis aligned through the center of the screw head to prevent head tilting (Figure 2).

Cyclic sinusoidal loading (0.5 Hz) began at 50-100 *N*, simulating physiological walking loads. Peak force increased 1.0 *N* per cycle (Locati protocol) to avoid degeneration effects. Failure was defined as 5.4 mm screw head displacement (∼20° tilt). Both sides were tested consecutively; testing order was alternated.

### Measured Parameters

Fatigue failure force (at 5.4 mm displacement) and construct stiffness were recorded at the start, at 50% of cycles, and at failure. Data were analyzed in Microsoft® Excel (v16.86, Redmond, WA, USA).

### Statistical Analysis

GraphPad Prism v9.5.1 (GraphPad Software, San Diego, CA, USA) was used. Significance was set at *P* = .05. Shapiro-Wilk tested normality; Levene’s test assessed variance homogeneity. Two-way ANOVA analyzed stiffness; fatigue loads were compared via *t*-tests.

## Results

### Specimen

There was no significant difference in gender distribution between the osteoporotic and healthy bone specimens (n.s., Fisher’s Exact test). VBMD of bone-healthy specimens was 131.7 ± 9.3 mg/cm^3^, while that of osteoporotic specimens was 68.3 ± 13.3 mg/cm^3^. The mean age of donors in the bone-healthy group was 57.8 ± 5.0 years, compared to 84.7 ± 4.3 years in the osteoporotic group. Both vBMD and age differed significantly between groups, with lower values observed in the osteoporotic group (*P* < .001).

### Fatigue Load and Cycles

**Figure 3.**
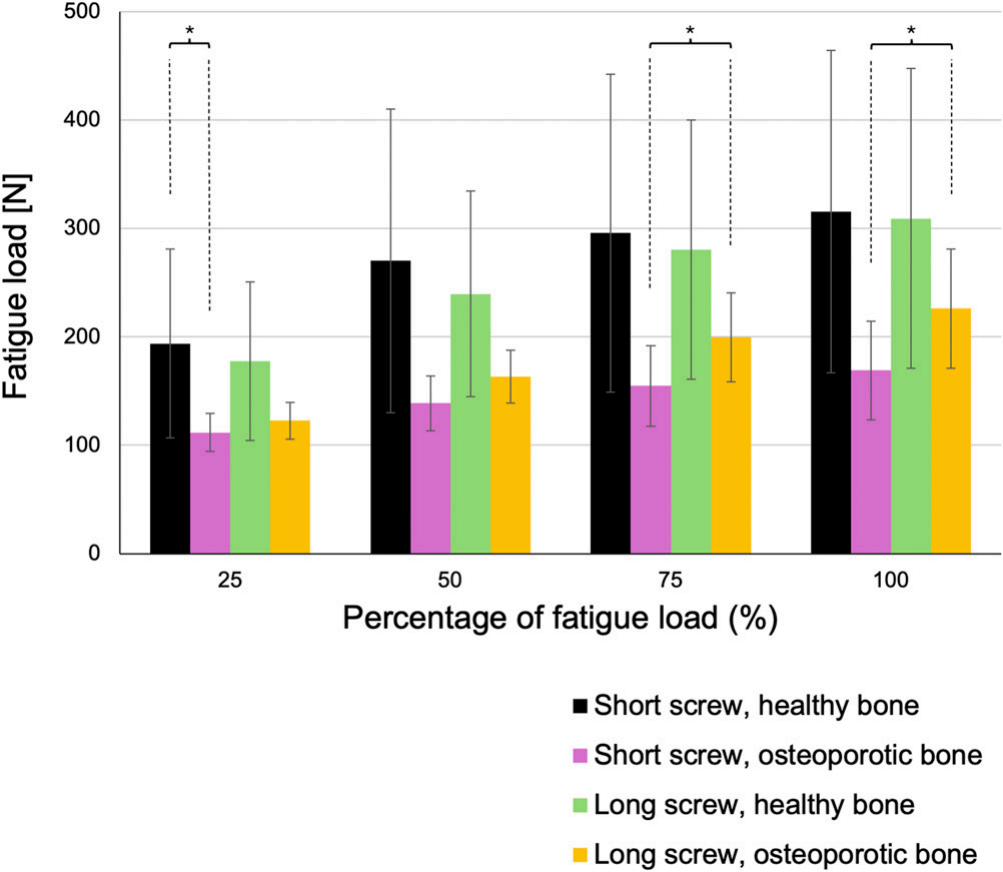
Fatigue load. In osteoporotic vertebral bodies, long screws withstood significantly greater cyclic-fatigue loads than did short screws

The mean fatigue load was 315.6 ± 148.7 *N* for short screws in vertebrae with healthy bone, 309.0 ± 138.3 *N* for long screws in vertebrae with healthy bone, 175.1 ± 45.5 *N* for short screws in osteoporotic vertebrae, and 230.9 ± 55.0 *N* for long screws in osteoporotic vertebrae. Long screws in osteoporotic vertebrae demonstrated a significantly higher fatigue load compared to short screws in osteoporotic vertebrae (*P* = .045). Furthermore, at 75% of the fatigue load, long screws in osteoporotic vertebrae sustained significantly higher loads than short screws in osteoporotic vertebrae (*P* = .049). At 25% of the fatigue load, short screws in healthy vertebrae supported significantly higher loads than those in osteoporotic vertebrae (Figure 3).

**Figure 4.**
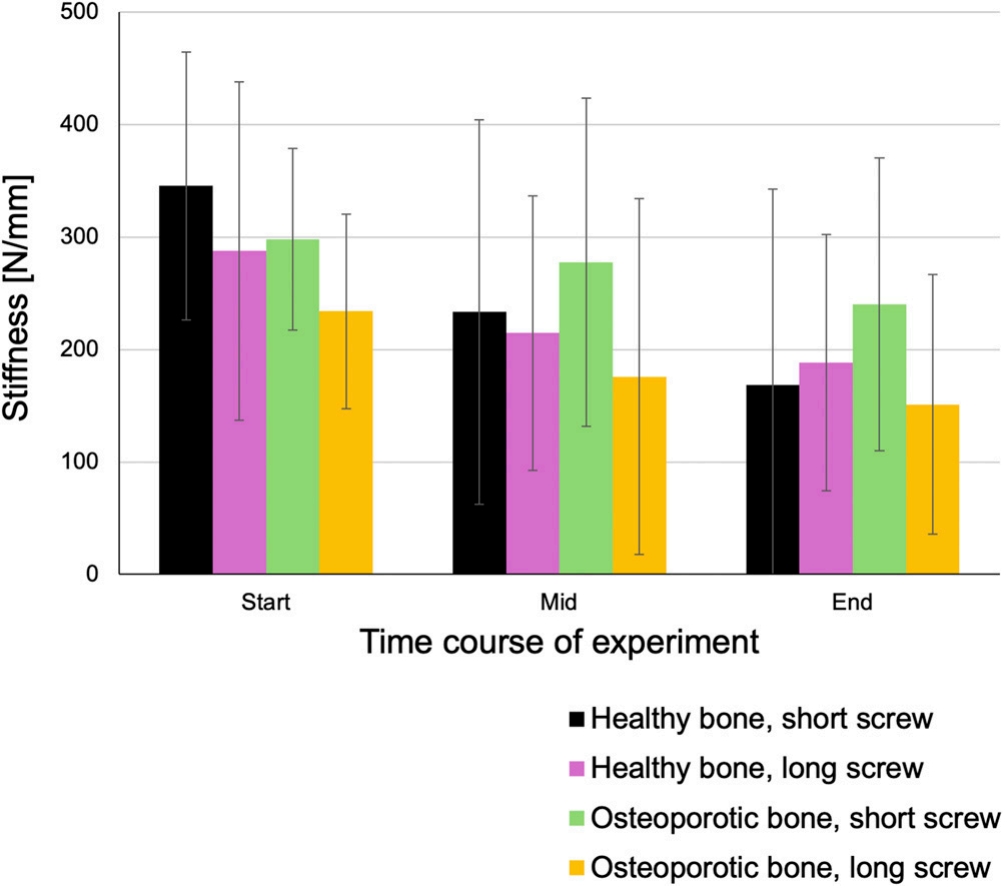
Cycles. Short screws inserted into osteoporotic vertebrae reached fatigue failure after significantly fewer loading cycles than both long screws in osteoporotic bone and short screws in healthy vertebrae

The average number of cycles at fatigue load was 2278 ± 1263 for short screws in vertebrae with healthy bone, 2446 ± 1365 for long screws in healthy bone vertebrae, 1071 ± 389 for short screws in osteoporotic vertebrae, and 1614 ± 553 for long screws in osteoporotic vertebrae. Short screws in osteoporotic vertebrae exhibited a significantly lower cycle count at fatigue failure compared to both long screws in osteoporotic vertebrae (*P* = .047) and short screws in healthy bone vertebrae (*P* = .049). This trend was consistent across 75%, 50%, and 25% of the fatigue load (Figure 4).

### Stiffness

**Figure 5.**
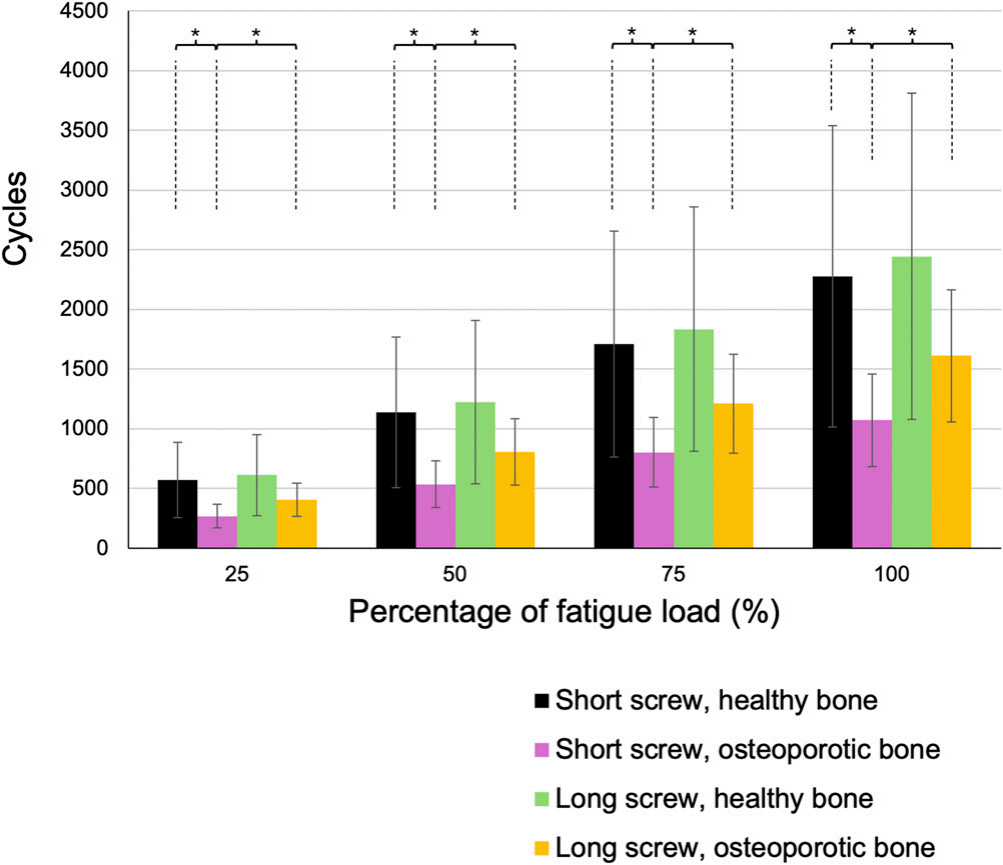
Stiffness. There were no statistically significant differences in stiffness between the screw groups

Final stiffness values were 168.7 ± 174.1 N/mm for short screws in healthy vertebrae, 188.3 ± 113.9 N/mm for long screws in healthy vertebrae, 240.2 ± 130.3 N/mm for short screws in osteoporotic vertebrae, and 151.2 ± 115.5 N/mm for long screws in osteoporotic vertebrae. No statistically significant differences in stiffness were observed among the groups (Figure 5).

## Discussion

The precise location of pedicle screw hold remains unclear due to conflicting results from biomechanical studies. In particular, the question of whether the site of pedicle screw anchorage differs between osteoporotic and non-osteoporotic (bone-healthy) vertebrae remains unanswered. The present findings refine the current understanding of pedicle screw anchorage by demonstrating distinct fixation patterns depending on bone quality.

In vertebrae with normal bone density, screw stability was nearly entirely achieved within the pedicle. The addition of screw length into the vertebral body did not enhance fatigue resistance, indicating that the pedicle alone provides sufficient cortical and cancellous engagement for long-term fixation. In osteoporotic vertebrae, overall screw stability was significantly reduced, reflecting the diminished structural competence of both pedicular and vertebral trabecular bone. While approximately three quarters of the screw hold was still provided by the pedicle, extending the screw into the vertebral body yielded a clear and statistically significant gain in fatigue strength compared with shorter screws confined to the pedicle. This suggests a shift in the relative contribution to screw hold—from a predominantly pedicular fixation in healthy bone toward a more balanced load sharing between pedicle and vertebral body as bone quality decreases.

These results complement and extend the findings of Hirano, Hasegawa, Takahashi, Uchiyama, Hara, Washio, Sugiura, Yokaichiya and Ikeda,^9^ who identified the cortical wall of the pedicle as the primary determinant of screw fixation strength but found no significant influence of bone mineral density. While their static pullout experiments characterized the initial fixation provided by the pedicle cortex, the present study, employing cyclic fatigue loading, reflects progressive mechanical degradation under repetitive stress. In this dynamic context, reduced bone quality appears to redistribute load transfer toward the vertebral body, making screw length a critical factor for maintaining anchorage in osteoporotic bone.

These findings contrast with prior anatomical and mechanical studies. Misenhimer, Peek, Wiltse, Rothman and Widell^6^ concluded that the pedicle itself is not the main contributor to screw fixation because oversized screws can cause cortical deformation before breach. In the present study, screw diameters were adapted to the inner cortical diameter, thereby preventing cortical damage and allowing undistorted assessment of pedicular hold.

Similarly, Defino and Vendrame^7^ concluded that cancellous bone, rather than the cortical structure of the pedicle, plays a more significant role in screw fixation, based on computed tomography analysis of screw trajectories. These earlier studies relied on indirect indicators—such as deformation or imaging findings—to infer the location of screw hold. In contrast, our study directly compares the fatigue resistance of short vs long screws in both osteoporotic and bone-healthy vertebrae. Although this remains an indirect approach (since direct measurement would require pressure sensors along the screw trajectory), it offers a more comparative and targeted analysis than previous investigations.

Contrary to the findings of Defino and Vendrame^7^ Misenhimer, Peek, Wiltse, Rothman and Widell,^6^ Cornaz, Farshad and Widmer^8^ employed a semi-direct approach by segmenting pedicle screws within sliced vertebrae. They reported that at low cyclic loading amplitudes, both the pedicle and vertebral body contributed equally to screw fixation, independent of bone quality. However, at higher cyclic amplitudes—particularly in osteoporotic bone—the pedicle’s contribution increased. They proposed that at low loads, the trabecular bone of the vertebral body offers greater support than that of the pedicle, whereas at higher loads (eg, 500 *N*), pedicle support becomes more prominent. This effect was especially pronounced in osteoporotic specimens.

While our results confirm a relevant pedicular contribution, we observed that this predominance is present mainly in bone-healthy vertebrae. In osteoporotic bone, the vertebral body plays a comparatively greater role, consistent with the notion of load redistribution due to reduced cortical support. Methodological differences may explain these contrasts: Cornaz, Farshad and Widmer^8^ used uniform 5 × 55 mm screws, whereas our study employed individualized diameters based on pedicle morphology, providing a more anatomically optimized and realistic model.

Throughout the experiments, stiffness did not significantly differ between screw lengths or bone qualities, which is consistent with expectations given that stiffness measurements were referenced to the applied force. Nonetheless, the number of cycles completed by each specimen varied considerably, with screws in healthy bone enduring a significantly higher peak load by the end of testing compared to those in osteoporotic bone.

From a clinical perspective, our findings suggest that in osteoporotic vertebrae, longer screws engaging the vertebral body can partially compensate for reduced cortical support and should be preferred when cement augmentation is undesirable. Although cement augmentation has been shown to substantially enhance screw fixation in osteoporotic bone,^12^ it carries known risks such as cement leakage and embolism. In contrast, in bone-healthy vertebrae, screw hold is primarily achieved within the pedicle; thus, shorter screws are sufficient and safer, avoiding potential anterior cortical breach or vascular injury.

In summary, this study demonstrates that in vertebrae with normal bone density, pedicle screws achieve fixation primarily within the pedicle. An exclusive fixation within the pedicle cannot be demonstrated by this study, as the short screws, with a length of 35 mm, may also engage approximately 1 cm of the vertebral body. In osteoporotic bone, overall stability decreases, and the relative contribution of the vertebral body to screw hold increases. Consequently, long screws provide a measurable and clinically relevant advantage in osteoporotic vertebrae, whereas in bone-healthy vertebrae, extending screw length confers no additional benefit and may pose unnecessary risk.

This study has several limitations. First, absence of in vivo biological conditions, including bone remodeling, osseointegration, and the physiological fluid environment, limits the generalizability of these cadaveric findings. Given the cadaveric setup, small sample size, and simplified loading conditions, the results should be interpreted primarily as mechanistic insights rather than definitive clinical guidance. Second, although cyclic fatigue (toggle) testing is widely regarded as the preclinical gold standard for evaluating pedicle screw performance,^13^ other testing modalities such as pull-out testing might have yielded different results. Third, while our comparison of long and short screws provides a more targeted evaluation than previous studies, it still constitutes an indirect method of assessing the anatomical source of screw hold. A direct measurement would require pressure sensors distributed along the screw trajectory. Nonetheless, compared to prior investigations,^6,8,13^ our method offers a more direct comparative analysis of screw hold localization by assessing mechanical performance across different screw lengths and bone qualities.

## References

[1] Defino H Galbusera F Wilke HJ . Pedicle screw fixation and design. In: Boden SD , ed. Lumbar Spine Online Textbook; 2025. chap 11, p. 9.

[2] Carmouche JJ Molinari RW Gerlinger T Devine J Patience T . Effects of pilot hole preparation technique on pedicle screw fixation in different regions of the osteoporotic thoracic and lumbar spine. J Neurosurg Spine. 2005;3(5):364-370. doi:10.3171/spi.2005.3.5.0364 16302630

[3] Chatzistergos PE Sapkas G Kourkoulis SK . The influence of the insertion technique on the pullout force of pedicle screws: an experimental study. Spine. 2010;35(9):E332-E337. doi:10.1097/BRS.0b013e3181ba0b0c 20150834

[4] Maiman DJ Pintar FA Yoganandan N , et al. Pull-out strength of caspar cervical screws. Neurosurgery. 1992;31(6):1097-1101. doi:10.1227/00006123-199212000-00016 1470320

[5] Ruland CM McAfee PC Warden KE Cunningham BW . Triangulation of pedicular instrumentation. A biomechanical analysis. Spine. 1991;16(6 Suppl):S270-S276. doi:10.1097/00007632-199106001-00019 1862424

[6] Misenhimer GR Peek RD Wiltse LL Rothman SL Widell EH Jr . Anatomic analysis of pedicle cortical and cancellous diameter as related to screw size. Spine. 1989;14(4):367-372. doi:10.1097/00007632-198904000-00004 2718038

[7] Defino HL Vendrame JR . Role of cortical and cancellous bone of the vertebral pedicle in implant fixation. Eur Spine J. 2001;10(4):325-333. doi:10.1007/s005860000232 11563619 PMC3611516

[8] Cornaz F Farshad M Widmer J . Location of pedicle screw hold in relation to bone quality and loads. Front Bioeng Biotechnol. 2022;10:953119. doi:10.3389/fbioe.2022.953119 36118575 PMC9478651

[9] Hirano T Hasegawa K Takahashi HE , et al. Structural characteristics of the pedicle and its role in screw stability. Spine. 1997;22(21):2504-2510. 9383856 10.1097/00007632-199711010-00007

[10] Weiser L Huber G Sellenschloh K , et al. Rescue augmentation: increased stability in augmentation after initial loosening of pedicle screws. Glob Spine J. 2021;11(5):679-685. doi:10.1177/2192568220919123 PMC8165920 32875910

[11] Weiser L Sellenschloh K Puschel K , et al. Cortical threaded pedicle screw improves fatigue strength in decreased bone quality. Eur Spine J. 2021;30(1):128-135. doi:10.1007/s00586-020-06593-3 32940741

[12] Song Z Zhou Q Jin X Zhang J . Cement-augmented pedicle screw for thoracolumbar degenerative diseases with osteoporosis: a systematic review and meta-analysis. J Orthop Surg Res. 2023;18(1):631. doi:10.1186/s13018-023-04077-w 37641101 PMC10464480

[13] Liebsch C Zimmermann J Graf N Schilling C Wilke HJ Kienle A . In vitro validation of a novel mechanical model for testing the anchorage capacity of pedicle screws using physiological load application. J Mech Behav Biomed Mater. 2018;77:578-585. doi:10.1016/j.jmbbm.2017.10.030 29096123

